# Needle biopsy compared with surgical biopsy: pitfalls of small biopsy in histologial diagnosis of IgG4-related disease

**DOI:** 10.1186/s13075-021-02432-y

**Published:** 2021-02-10

**Authors:** Yanying Liu, Fei Yang, Xiying Chi, Yuxin Zhang, Jiangnan Fu, Wenjie Bian, Danhua Shen, Zhanguo Li

**Affiliations:** 1 Department of Rheumatology and Immunology, Peking University People’s Hospital, Beijing, 100044 China; 2 Department of Pathology, Peking University People’s Hospital, Beijing, 100044 China

**Keywords:** IgG4-related disease, Needle biopsy, Surgical biopsy, Salivary gland, Pathology consensus

## Abstract

**Objective:**

The growing utilization of needle biopsy has challenged the current pathology consensus of IgG4-related disease (IgG4-RD). The aims of this study were to identify the histological characteristics of needle biopsy and surgical specimens and evaluate the ability of needle biopsy in histological diagnosis of IgG4-RD.

**Methods:**

Biopsies from patients who were referred to as IgG4-RD by the 2019 ACR/EULAR IgG4-RD classification criteria in Peking University People’s Hospital from 2012 to 2019 were re-evaluated. Typical histological features and diagnostic categories were compared between needle biopsy and surgical biopsy.

**Results:**

In total, 69 patients met the 2019 ACR/EULAR classification criteria and 72 biopsies of them were re-evaluated. All cases showed lymphoplasmacytic infiltrate, while storiform fibrosis and obliterative phlebitis were only present in 35 (48.6%) and 23 (31.9%) specimens, respectively. Storiform fibrosis was more likely to be seen in retroperitoneum lesion (*P* = 0.033). Surgical biopsy showed significantly higher IgG4+ plasma cells/high-power field (IgG4/HPF) count (*P* < 0.01) and higher proportion of IgG4/HPF > 10 (*P* < 0.01). No significant difference was observed with regard to the ratio of IgG4+ plasma cells/IgG+ plasma cells (IgG4/IgG) (*P* = 0.399), storiform fibrosis (*P* = 0.739), and obliterative phletibis (*P* = 0.153). According to the 2011 comprehensive diagnostic criteria, patients who performed a needle biopsy were less likely to be probable IgG4-RD (*P* = 0.045). Based on the 2011 pathology consensus, needle biopsy was less likely to be diagnosed as IgG4-RD (*P* < 0.01), especially to be highly suggestive IgG4-RD (*P* < 0.01). Only 1/18 (5.6%) needle salivary specimens fulfilled the cutoff of IgG4/HPF > 100, which was significantly less than 15/23 (65.2%) of surgical ones (*P* < 0.01).

**Conclusions:**

Needle biopsy shows an inferiority in detecting IgG4/HPF count but not in IgG4/IgG ratio, storiform fibrosis, and obliterative phlebitis. Compared with surgical samples, needle biopsy is less likely to obtain a histological diagnosis of IgG4-RD. A different IgG4/HPF threshold for needle biopsy of the salivary glands may be considered.

**Supplementary Information:**

The online version contains supplementary material available at 10.1186/s13075-021-02432-y.

## Introduction

IgG4-related disease (IgG4-RD) is a newly recognized fibroinflammatory condition characterized by tumefactive lesions involved in multiple sites; often but not always, the elevated serum IgG4 concentration; and a dense lymphoplasmacytic infiltrate rich in IgG4-positive plasma cells [1]. Comprehensive diagnostic criteria were established in 2011, dividing IgG4-RD into possible, probable, and definite cases based on the clinical, serological, and pathological evidence [2] (Supplementary Table 1). At the same time, a pathology consensus for IgG4-RD was published for histopathological diagnosis based on histological traits and immunohistochemical features [3] (Supplementary Table 2). Despite the importance of biopsy in excluding many mimickers, biopsy is not always easily accessible or acceptable by patients in many cases. In the context of that, the 2019 ACR/EULAR classification criteria were codified, allowing the judgment of IgG4-RD in the absence of a biopsy [4]. Nevertheless, the complexity has constrained the utilization of the classification criteria for clinical purpose. Therefore, comprehensive diagnostic criteria that largely depend on pathology are still dispensable in clinical practice. Recently, amplified utilization of needle biopsy has been challenging the pathology consensus since current pathology recommendations are largely based on resection specimens [5]. However, to the best of our knowledge, there have been no studies comparing the ability of needle biopsy and open surgical biopsy in overall IgG4-RD spectrum. And few literatures have re-evaluated the 2011 pathology consensus.

We performed a comparison of needle biopsy and surgical biopsy in terms of histological features and diagnostic categories and re-evaluated the 2011 pathology consensus based on 72 biopsied specimens (either needle or surgical biopsy) from 69 patients who underwent biopsy and were referred to as IgG4-RD by the 2019 ACR/EULAR IgG4-RD classification criteria in Peking University People’s Hospital from 2012 to 2019 [4]. The aim of this study was to identify the histological characteristics of different biopsy specimens and evaluate the diagnostic ability of needle biopsy.

## Materials and methods

Patients who underwent biopsy (either needle biopsy or surgical resection) in Peking University People’s Hospital and were histopathologically suggested or suspected as IgG4-RD from 2012 to 2019 were enrolled. All patients provided written informed consent. The following data were collected from the medical records: gender, age of disease onset, and baseline serum IgG4 concentration. The disease activity reflected by IgG4-related disease responder index (IgG4-RD RI) was calculated by two independent physicians and mean value was taken [6].

Two pathologists who were blind to the sample information worked independently and re-evaluated the hematoxylin and eosin (H&E)–stained, IgG4 and IgG stained slides of enrolled patients. The degree of fibrosis and lymphoplasmacytic infiltrates was assessed. Typical histopathological characteristics of storiform fibrosis and obliterative phlebitis were recorded. Three 40× fields with the highest number of IgG4 plasma cells were calculated, and the average number was recorded. The same three fields were counted for the IgG4/IgG ratio [2, 3]. In cases where the pathologists disagreed, specimens were evaluated in tandem and a diagnosis was assigned based on consensus. Cases would be excluded when a consensus could not be reached. Thereafter, criteria score was calculated based on the clinical, serological, and pathological evidence according to the 2019 ACR/EULAR classification criteria. Cases who failed to meet the classification criteria with a score less than 20 were excluded.

Quantitative variables with non-normally distribution were presented as medians and interquartile range (IQR) and were compared with Mann-Whitney test. Quantitative variables with normally distribution were presented as mean ± standard deviation (SD) and were compared with Student’s *t* test. Categorical variables were assessed with the chi-squared test or Fisher’s exact test, as appropriate. *P* values were adjusted with Bonferroni method when comparing multiple categorical variables in pairs. *P* values < 0.05 were considered statistically significant. All statistical analyses were performed by SPSS version 25.0. All figures were made by the Origin 2018.

This study was approved by the Medical Ethics Committee of Peking University People’s Hospital (Beijing, China).

## Results

### Clinical characteristics of the 69 patients and pathological features of the 72 specimens

In total, 69 patients met the 2019 ACR/EULAR criteria, whose classification criteria scores were all above 20 and the median score was 39.5 (31.3, 47.3). Forty-four of the 69 patients (61.1%) were males, and the median age was 56.0 (50.3, 64.0). The median IgG4-RD RI was 4.0, and the median serum IgG4 concentration was 770 mg/dl. Three patients underwent two biopsies in different organs, and 72 specimens in all were re-evaluated (Table 1).

**Table 1:** Clinical and pathological characteristics of the patients

Characteristics	Value
Age, median (IQR)	56.0 (50.3, 64.0)
Gender, male, *n* (%)	44 (61.1)
Serum IgG4 (mg/dl), median (IQR)	770.0 (230.8, 1870.0)
IgG4-RD RI	4.0 (2.0, 6.3)
Serum IgG4, *n* (%)	
< 1 ULN	14 (19.4)
1 ~ 2 ULN	11 (15.3)
2 ~ 5 ULN	14 (19.4)
≥ 5 ULN	32 (44.4)
Missing value	1 (1.4)
Biopsy method, *n* (%)	
Needle biopsy	24 (33.3)
Surgical biopsy	48 (66.7)
Biopsy organ, *n* (%)	
Meninges	1 (1.4)
Lacrimal gland	6 (8.3)
Salivary gland	41 (56.9)
Lymph node	8 (11.1)
Lung	2 (2.8)
Pleura	1 (1.4)
Pancreas	2 (2.8)
Bile duct	1 (1.4)
Retroperitoneum	9 (12.5)
Prostate	1 (1.4)
IgG4/HPF, median (IQR)	80.0 (50.0, 130.0)
IgG4/HPF, *n* (%)	
> 10	66 (91.7)
≤ 10	3 (4.2)
Indetermined	3 (4.2)
IgG4/IgG (%), mean ± SD	67.5 ± 23.3
IgG4/IgG, *n* (%)	
> 40%	62 (86.1)
≤ 40%	9 (12.5)
Indetermined	1 (1.4)
Lymphoplasmacytic infiltrate	72 (100.0)
Storiform fibrosis, *n* (%)	35 (48.6)
Obliterative phlebitis, *n* (%)	23 (31.9)
2019 ACR/EULAR Criteria Score, median (IQR)	39.5 (31.3, 47.3)
2011 comprehensive diagnostic criteria, *n* (%)	
Possible	11 (15.3)
Probable	14 (19.4)
Definite	47 (65.3)
2011 Pathology diagnostic consensus, *n* (%)	
Insufficient	30 (41.7)
Probable	18 (25.0)
Highly suggestive	24 (33.3%)

Salivary gland (41/72, 56.9%) was the most common biopsy site. Other involved organs included retroperitoneum (9/72, 12.5%), lymph nodes (8/72, 11.1%), lacrimal gland (6/72, 8.3%), lung (2/72, 2.8%), pancreas (2/72, 2.8%), meninges (1/72, 1.4%), pleura (1/72, 1.4%), bile duct (1/72, 1.4%), and prostate (1/72, 1.4%). According to the 2011 comprehensive diagnostic criteria, 11 (15.3%) of the patients were possible, 14 (19.4%) were probable, and 47 (65.3%) were definite IgG4-RD. For patients who performed two biopsies, the diagnostic categories were identical. According to the 2011 pathology consensus, 30 (41.7%) of the biopsies were insufficient, 18 (25.0%) were probable, and 24 (33.3%) were highly suggestive IgG4-RD (Table 1).

**Fig. 1:**
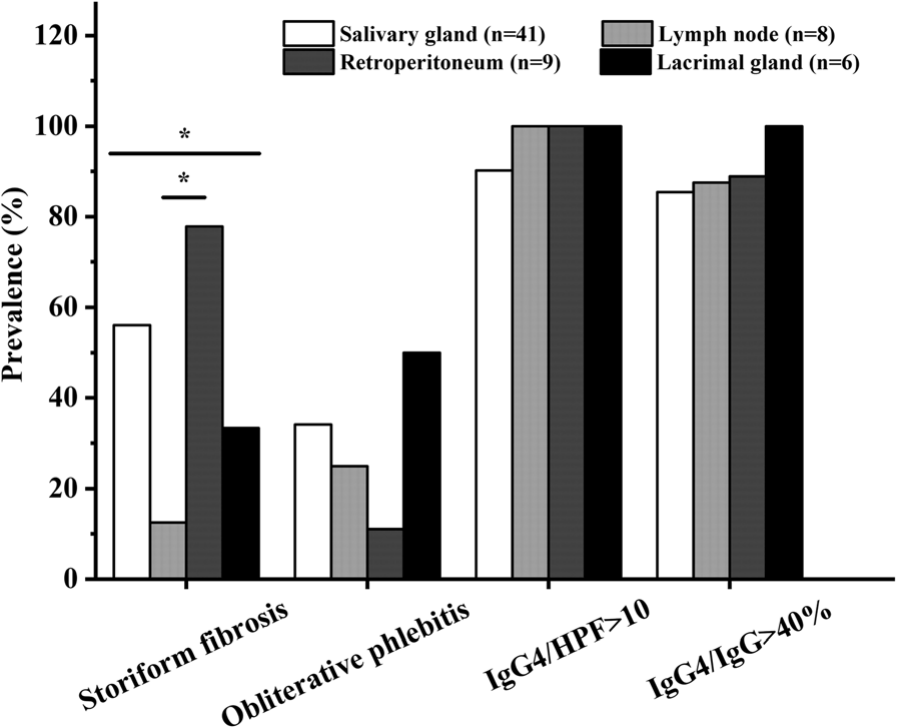
Histological characteristics of different organs among patients with IgG4-RD. *represents *P* < 0.05

Dense lymphoplasmacytic infiltrate was ubiquitous regardless of the biopsy sites or methods. The median IgG4/HPF count was 80.0 (50.0, 130.0), and the average IgG4/IgG ratio was 67.5 ± 23.3%. Retroperitoneum specimen tended to show less IgG4/HPF count than other organs, even though no significant difference was found (*P* = 0.177). Sixty-two (86.1%) and 66 (91.7%) biopsies showed IgG4/IgG > 40% and IgG4/HPF > 10, respectively. Storiform fibrosis and obliterative phlebitis were comparatively uncommon and were only present in 35 (48.6%) and 23 (31.9%) specimens, respectively (Table 1). No organ specificity of obliterative phlebitis was observed among different organs (*P* = 0.446), while storiform fibrosis was more likely to be seen in retroperitoneum lesion (*P* = 0.033) (Fig. 1, Supplementary Table 3).

### Needle biopsy versus surgical biopsy

**Fig. 2:**
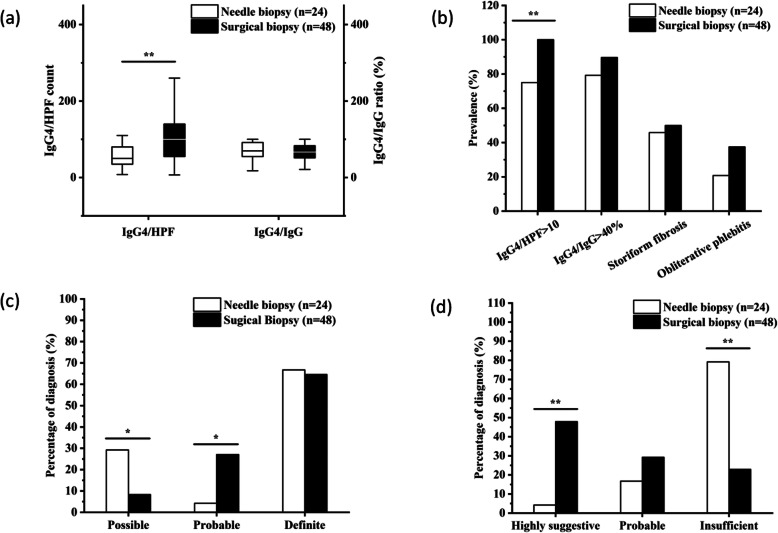
Comparison of needle biopsy and surgical biopsy in 72 samples of patients diagnosed as IgG4-RD. **a** The comparison of IgG4/HPF count and the ratio of IgG4/IgG in different biopsy method groups. **b** The comparison of histopathological features between the two groups. **c** The difference of the diagnostic coincidence rates between the two groups with 2011 comprehensive diagnostic criteria. **d** The difference of the diagnostic coincidence rates between the two groups with histological diagnosis categories. *P < 0.05, **P < 0.01

48/72 (66.7%) specimens were surgical biopsy. IgG4-RD RI (*P* = 0.711) demonstrated no difference between the two groups. No significant discrepancy was found between needle biopsy and surgical biopsy in terms of IgG4/IgG ratio (*P* = 0.438), proportion of IgG4/IgG > 40% (*P* = 0.399), storiform fibrosis (*P* = 0.739), and obliterative phlebitis (*P* = 0.153). However, needle specimens tended to show significantly less IgG4/HPF count (*P* = 0.003) and lower proportion of IgG4/HPF > 10 (*P* = 0.001) than needle biopsy. Compatible with that, according to the 2011 comprehensive diagnostic criteria, patients who performed a needle biopsy were less likely to be probable IgG4-RD (*P* = 0.045) while serum IgG4 showed no significant difference (*P* = 0.618). Consistently, based on the 2011 pathology consensus, needle biopsy was less likely to be histologically identified as IgG4-RD (*P* < 0.001), especially to be highly suggestive IgG4-RD (*P* < 0.001) (Fig. 2, Supplementary Table 4).

Given the limited samples obtained from the meninges, lung, pleura, pancreas, bile duct, and prostate, we excluded these entities for repetitive analysis. Compatible with the results above, no difference was observed pertaining to IgG4-RD RI (*P* = 0.939), IgG4/IgG ratio (*P* = 0.437), prevalence of IgG4/IgG > 40% (*P* = 1.000), storiform fibrosis (*P* = 0.711), and obliterative phlebitis (*P* = 0.191). However, IgG4/HPF count (*P* < 0.001) and the proportion of IgG4/HPF > 10 (*P* = 0.008) in surgical biopsy were dramatically higher than that in surgical biopsy (*P* < 0.001). These data suggested that needle biopsy might be less capable to capture full IgG4 profile and thus insufficient IgG4/HPF count, which led to less possibility of histological identification based on the 2011 pathology consensus statement.

### Re-evaluation of 2011 pathology consensus

According to the 2011 pathology consensus, 24 of the 72 IgG4-RD (33.33%) were highly suggestive, 18 (25.00%) were probable, and 30 (41.67%) were insufficient to be identified as IgG4-RD. Given that all the 72 specimens showed at least one of the three pathological features (lymphoplasmacytic infiltrate), all of these insufficient biopsies failed to fulfill either required IgG4/HPF cutoff point or IgG4/IgG > 40%. Even though IgG4/HPF > 10 had been proposed as one component of a comprehensive diagnostic panel, the pathology criteria recommended a set of IgG4/HPF threshold that was specific to each organ, from 10 to 200. In the 30 (41.67%) insufficient IgG4-RD specimens, 7 (9.72%) showed IgG4/IgG ≤ 40% and 29 (40.28%) showed less IgG4/HPF count than the cutoff value as recommended by the consensus (Table 2).

**Table 2:** Histological diagnosis according to 2011 pathological consensus of 72 IgG4-RD specimens

	*n*^a^	*N*^b^	*n*/72^c^ (%)	*n*/*N*^d^ (%)
Highly suggestive	24	–	33.33	–
Probable	18	–	25.00	–
Insufficient	30	–	41.67	–
IgG4/IgG < 40%	7	–	9.72	–
Salivary gland	4	41	5.55	9.76
Retroperitoneum	1	9	1.39	11.11
Lymph node	1	8	1.39	12.50
Pancreas	1	2	1.39	50.00
IgG4/HP < cutoff value	29	–	40.28	–
Salivary gland	23	41	31.94	56.10
Lymph node	2	8	2.78	25.00
Lung	1	2	1.39	50.00
Lacrimal gland	1	6	1.39	16.67
Pleura	1	1	1.39	100.00
Pancreas	1	2	1.39	50.00

^a^*n* represents the number of cases

^b^*N* represents the number of biopsies taken from the same organ

^c^Proportion in all the 72 biopsies

^d^Proportion in all biopsies of the same organ

**Fig. 3:**
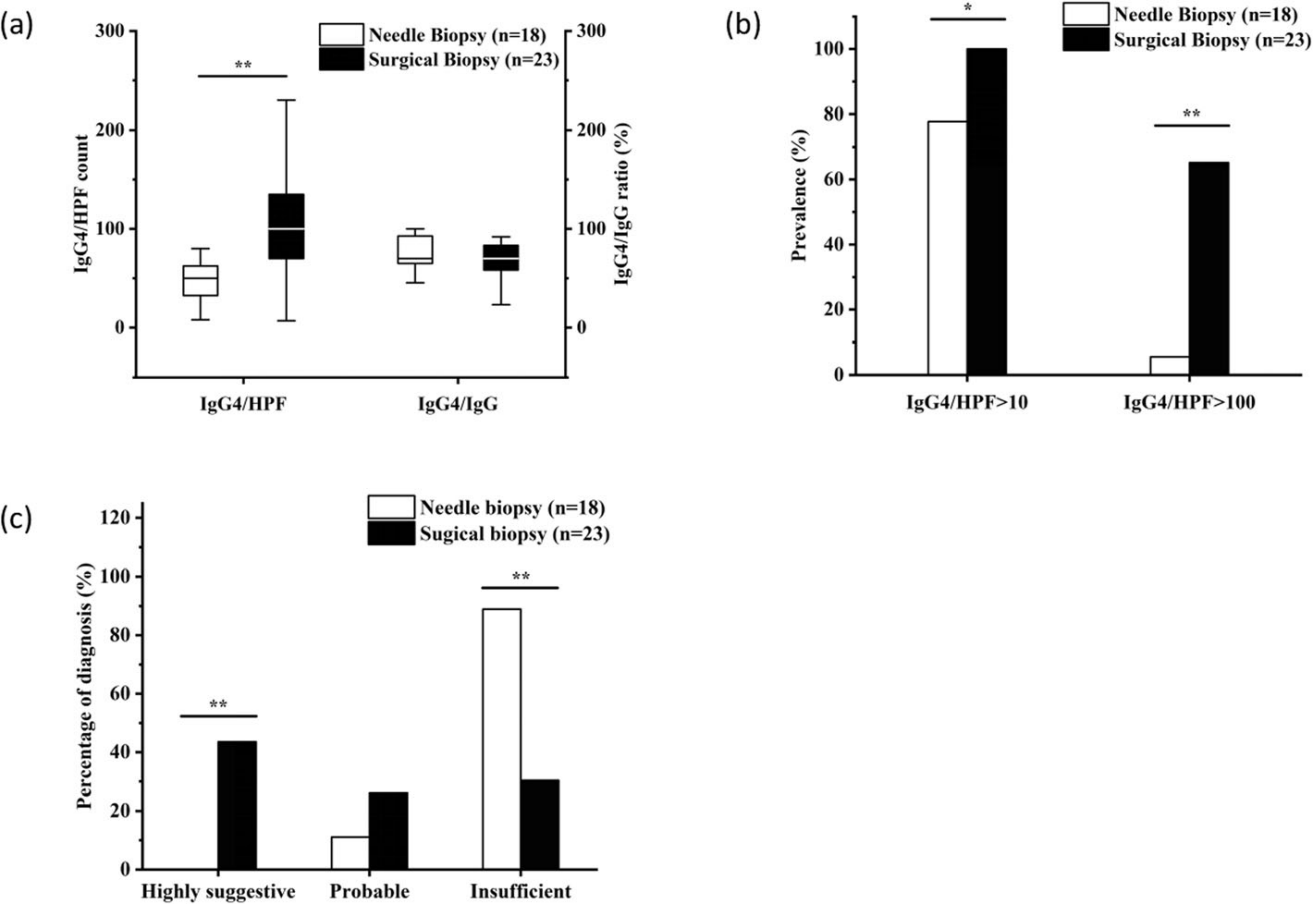
Comparison of needle biopsy and surgical biopsy in 41 salivary gland samples. **a** The comparison of IgG4/HPF count and the ratio of IgG4/IgG in different biopsy method groups. **b** The comparison of the proportions of IgG4/HPF > 10 and IgG4/HPF > 100 between the two groups. **c** The difference of the diagnostic coincidence rates between the two groups with histological diagnosis categories. *P < 0.05, **P < 0.01

In the 29 (40.28%) specimens with insufficient IgG4/HPF count, 23 (31.94%) were taken from salivary glands, 2 (2.78%) were from lymph nodes, and 1 (1.39%) was from the lung, lacrimal gland, pleura, and pancreas, respectively. The cutoff IgG4/HPF value of the salivary gland recommended by the pathology consensus was 100/HPF. Nevertheless, in this cohort, only 16 out of 41 (39.02%) salivary samples had met this threshold, and 15 of them were surgical specimens while only 1 was needle biopsy (Table 2). To better identify the diagnostic efficiency of different biopsy methods according to the pathology consensus, the salivary gland was chosen as the only organ specimen (Fig. 3).

Consistent with the results above, no significant discrepancy was found between two groups in terms of IgG4-RD RI (*P* = 0.695), IgG4/IgG (*P* = 0.720), and proportion of IgG4/IgG > 40% (*P* = 1.000). But surgical biopsy tended to show significantly higher IgG4/HPF count (*P* < 0.001) in salivary samples. No more than 1/18 (5.6%) salivary needle specimens met the cutoff value of IgG4/HPF > 100, which was significantly less than 15/23 (65.2%) of surgical ones (*P* < 0.001). Additionally, salivary needle biopsy was more likely to be neither highly suggestive nor probable IgG4-RD (*P* < 0.001). These data suggested that, compared with surgical biopsy, needle biopsy was hardly able to capture required IgG4/HPF count and thus be judged as IgG4-RD in salivary glands based on the pathology consensus (Fig. 3).

## Discussion

IgG4-RD is a chronic mass-forming fibroinflammatory disease that may be involved in multiple organs. Typical histopathological features of IgG4-RD include dense lymphoplasmacytic infiltrate, storiform fibrosis, and obliterative phlebitis. In this study, biopsy of IgG4-RD lesions ubiquitously showed lymphoplasmacytic infiltrate, but often lacking either storiform fibrosis or obliterative phlebitis. Furthermore, needle biopsy proved to be less capable of detecting IgG4/HPF count and was inferior in diagnosing IgG4-RD, especially in salivary gland lesions.

Storiform fibrosis and obliterative phlebitis are two typical features of IgG4-RD, and the histological appearance of them usually shows high specificity. However, in this study, merely 48.6% biopsies showed the storiform fibrosis and obliterative phlebitis were only present in 31.9% samples. Moreover, organ-specific differences including the absence of storiform fibrosis within lacrimal glands and lymph nodes, and the lower frequency of obliterative phlebitis in salivary glands, lymph nodes, and retroperitoneum were also observed. These results were in line with the previous studies [7, 8]. Furthermore, even though no significant discrepancy was found in this study, it had been suggested that storiform fibrosis and obliterative phlebitis might be scarcely detected in small samples like needle biopsy [3, 9]. These results suggest the importance of IgG4/HPF count and IgG4/IgG ratio in the judgment of IgG4-RD specimens when the storiform fibrosis and obliterative phlebitis are absent.

In the IgG4-RD pathology consensus, 3-tiered terminology (insufficient, probable, and highly suggestive) were endorsed for the histopathological judgment based on the three histological features (lymphoplasmacytic infiltrate, storiform fibrosis, and obliterative phlebitis), and immunohistochemical features (ratio of IgG4/IgG and IgG4/HPF count). In our cohort, nearly half of salivary specimens were defect of enough IgG4/HPF count for histological identification. Similarly, Andrew et al. reported that two thirds of lacrimal IgG4-RD cases failed to be diagnosed using a cutoff of IgG4/HPF > 100 as recommended by the consensus, but the difference between needle biopsy and surgical samples was not explored [10]. Even though some researchers have suggested performing a salivary gland biopsy when IgG4-RD is suspected [11, 12], in this study, however, most of the insufficient salivary samples were biopsied by needle, while most surgical samples still met the threshold of IgG4/HPF > 100. Indeed, in the 2011 pathology consensus, biopsy samples in organs like the lung, pancreas, bile duct, liver, and kidney have lower IgG4/HPF threshold than that of surgical ones. While the cutoff of IgG4/HPF count for other organs including the salivary gland, lacrimal gland, lymph node, pleura, retroperitoneum, aorta, and skin suggested by the consensus, as we have observed, are more suitable to surgical samples but less applicable to the small biopsy [3, 13]. It is noteworthy that setting the cutoff to IgG4/HPF > 10, surgical cases that meet this threshold are still significantly more than needle biopsies. Therefore, we suggest the superiority of surgical samples in a suspicious IgG4-RD salivary lesion. And a distinctive cutoff IgG4/HPF count other than 100/HPF for needle biopsy of salivary gland may be considered.

Despite the superiority of surgical biopsy in detecting IgG4/HPF count, IgG4/IgG ratio showed no divergence between two groups. Even though IgG4/IgG ratio was deemed as a more powerful tool than IgG4/HPF in establishing the diagnosis of IgG4-RD; however, in the absence of other corroborative findings, we are unable to accept IgG4/IgG > 40% itself as sufficient diagnostic evidence in the light of the pathology consensus [3]. This applies particularly to cases with low IgG4/HPF count. Therefore, surgical biopsy still shows obvious advantage as it does well in calculating both IgG4/HPF and IgG4/IgG.

The major issue of needle biopsy is represented by the inadequacy of the material obtained for histopathological evaluation and immunohistochemical tests, and thus incomplete characterization of a lymphoproliferative disorder [14, 15]. Besides, plasma cells crushed by an artifact tend to cause unsuccessful immunostaining in smaller samples [16]. The quality of a needle biopsy might also depend on the experience of the operator, the number of cores obtained from the lesion, the gauge of the needle we used and the ultrasound direction [17].

However, needle biopsy still has some advantages. Compared with surgical resection, needle biopsy is less invasive, less expensive, and usually with fewer long-term or transient complications, which have a high impact on the patient’s acceptance, especially when the biopsy of an internal viscera is needed. Moreover, even though open surgical biopsies allowed the adequate material for pathological evaluation, it is a non-targeted approach. Regions with restricted lesions may not represent the full features of the disorder. In contrast, ultrasound-guided needle biopsy is able to distinguish areas with different sonographic patterns and target the most suspicious lesions [17]. In addition, whenever any kind of neoplasm is possible, open biopsy is contraindicated since it may compromise patients’ outcome (e.g., by increasing the risk of tumor recurrence) [18, 19].

Improving the specificity of the pathology criteria by setting high-organ-specific cutoffs for IgG4 staining is essential. Even though we interpreted a lower IgG4 cutoff for needle biopsy on salivary gland, it remains to figure out whether needle biopsy is feasible or not for the diagnosis of IgG4-RD and if a different IgG4/HPF cutoff for needle biopsy might impair the specificity. Last but not least, despite the importance of pathology, additional clinical, serological, and radiological evidence is still indispensable for confirming the ultimate diagnosis of IgG4-RD. Patients who lacked one or more of the histological and immunohistochemical features of IgG4-RD may overlap with those showing definite features with regard to serum IgG4 levels, multiorgan involvement, and response to glucocorticoid therapy [5]. Even cases classified in the pathological category of insufficient IgG4-RD do not exclude the diagnosis thoroughly. For highly suggestive cases, the pathology criteria might not be infallible, either [20, 21]. Potential reasons might include sampling artifact, the effects of previous therapy, and progression to a fibrotic stage, etc. [3].

What should be emphasized is that, biopsies from the same organ of the same patient could be more representative. However, biopsy method was determined by the entity site and patients’ acceptability. Matched samples of both needle and surgical biopsy from the same patient were hardly to obtain. Only one patient in our cohort had matched specimens, whose surgical biopsy also showed slightly higher IgG4/HPF count. In spite of that, demographic characteristics and disease activity reflected by IgG4-RD RI demonstrated no significant disparity, suggesting the comparability of the two biopsy method groups and the reliability of our findings.

One limitation is that, the cohort of this study does not cover all the common lesions of IgG4-RD, and most of the samples were taken from the salivary glands. It is still unable to validate the diagnostic capability of needle biopsy with regard to calculating IgG4/HPF count when dealing with other organ samples. It remains to figure out whether a different diagnostic threshold of IgG4/HPF for other entities should be set.

## Conclusions

In conclusion, needle biopsy shows an inferiority in detecting IgG4/HPF count but not in IgG4/IgG ratio, storiform fibrosis, and obliterative phlebitis. Compared with surgical samples, needle biopsy is less likely to obtain a histological diagnosis of IgG4-RD. A different IgG4/HPF threshold for needle biopsy of the salivary glands may be considered.

## Supplementary Information

**Additional file 1:.** Supplementary Table 1. 2011 Comprehensive clinical diagnostic criteria. Supplementary Table 2. 2011 pathology consensus. Supplementary Table 3. Histological Characteristics in 4 biopsy organs of classified as IgG4-RD. Supplementary Table 4. Comparison of Needle Biopsy and Surgical Biopsy in 72 diagnosed IgG4-RD samples.

## Data Availability

Not applicable.

## Abbreviations

IgG4-RD: IgG4-related disease
IgG4/HPF: IgG4+ plasma cells/high power field
IgG4/IgG: The ratio of IgG4+ plasma cells/IgG+ plasma cells
IgG4-RD RI: IgG4-related disease responder index
H&E: Hematoxylin and eosin
IQR: Interquartile range
SD: Standard deviation

## References

[1] Stone JH, Zen Y, Deshpande V (2012). IgG4-related disease. N Engl J Med.

[2] Umehara H, Okazaki K, Masaki Y (2012). Comprehensive diagnostic criteria for IgG4-related disease (IgG4-RD), 2011. Mod Rheumatol.

[3] Deshpande V, Zen Y, Chan JKC (2012). Consensus statement on the pathology of IgG4-related disease. Mod Pathol.

[4] Wallace ZS, Naden RP, Chari S (2020). The 2019 American College of Rheumatology/European league against rheumatism classification criteria for IgG4-related disease. Ann Rheum Dis.

[5] Arora K, Rivera M, Ting DT (2019). The histological diagnosis of IgG4-related disease on small biopsies: challenges and pitfalls. Histopathology.

[6] Wallace ZS, Khosroshahi A, Carruthers MD (2018). An international multispecialty validation study of the IgG4-related disease responder index. Arthritis Care Res (Hoboken).

[7] Kamisawa T, Zen Y, Pillai S (2015). IgG4-related disease. Lancet.

[8] Zen Y, Nakanuma Y (2010). IgG4-related disease: a cross-sectional study of 114 cases. Am J Surg Pathol.

[9] Divatia M, Kim SA, Ro JY (2012). IgG4-related sclerosing disease, an emerging entity: a review of a multi-system disease. Yonsei Med J.

[10] Andrew N, Kearney D, Selva D (2013). Applying the consensus statement on the pathology of IgG4-related disease to lacrimal gland lesions. Mod Pathol.

[11] Takano K, Nomura K, Abe A (2016). Clinicopathological analysis of salivary gland tissue from patients with IgG4-related disease. Acta Otolaryngol.

[12] Takano K, Keira Y, Seki N (2014). Evaluation of submandibular versus labial salivary gland fibrosis in IgG4-related disease. Mod Rheumatol.

[13] Peuraharju E, Saarinen R, Aro K (2020). Sclerosing sialadenitis of the submandibular gland is rarely an immunoglobulin G4-related disease in the Finnish population. Mod Pathol.

[14] Kim HJ, Kim JS (2018). Ultrasound-guided core needle biopsy in salivary glands: a meta-analysis. Laryngoscope.

[15] Witt BL, Schmidt RL (2014). Ultrasound-guided core needle biopsy of salivary gland lesions: a systematic review and meta-analysis. Laryngoscope.

[16] Notohara K, Kamisawa T, Kanno A (2020). Efficacy and limitations of the histological diagnosis of type 1 autoimmune pancreatitis with endoscopic ultrasound-guided fine needle biopsy with large tissue amounts. Pancreatology.

[17] Zabotti A, Zandonella Callegher S, Lorenzon M, et al. Ultrasound-guided core needle biopsy compared with open biopsy: a new diagnostic approach to salivary gland enlargement in Sjogren's syndrome? Rheumatology (Oxford). 2020. 10.1093/rheumatology/keaa441. 10.1093/rheumatology/keaa441 32940706

[18] Atula T, Panigrahi J, Tarkkanen J (2017). Preoperative evaluation and surgical planning of submandibular gland tumors. Head Neck.

[19] Aro K, Valle J, Tarkkanen J (2019). Repeatedly recurring pleomorphic adenoma: a therapeutic challenge. Acta Otorhinolaryngol Ital.

[20] Deshpande V, Zane NA, Kraft S (2016). Recurrent mastoiditis mimics IgG4 related disease: a potential diagnostic pitfall. Head Neck Pathol.

[21] Taylor MS, Chougule A, Macleay AR (2019). Morphologic overlap between inflammatory myofibroblastic tumor and IgG4-related disease: lessons from next-generation sequencing. Am J Surg Pathol.

