# Supplementary material for: Needle biopsy compared with surgical biopsy: pitfalls of small biopsy in histologial diagnosis of IgG4-related disease
Source: Arthritis Res Ther. 2021 Feb 10;23:54. doi: 10.1186/s13075-021-02432-y (PMC7874654; doi:10.1186/s13075-021-02432-y)
Supplement: Supplementary file 1 — Additional file 1:. Supplementary Table 1. 2011 Comprehensive clinical diagnostic criteria. Supplementary Table 2. 2011 pathology consensus. Supplementary Table 3. Histological Characteristics in 4 biopsy organs of classified as IgG4-RD. Supplementary Table 4. Comparison of Needle Biopsy and Surgical Biopsy in 72 diagnosed IgG4-RD samples. [file 13075_2021_2432_MOESM1_ESM.docx]

Supplementary Table 1 2011 Comprehensive clinical diagnostic criteria

| **Comprehensive clinical diagnostic criteria** |
| --- |
| 1. Diffuse/localized swelling or masses in single or multiple organs  2. Elevated serum IgG4 concentrations (135 mg/dl)  3. Histopathologic examination shows:  (1) Marked lymphocyte and plasmacyte infiltration and fibrosis  (2) Ratio of IgG4+/IgG+ cells > 40% and IgG4+/HPF>10 |
| Definite: 1 + 2 + 3  Probable: 1 + 3  Possible: 1 + 2 |
| However, it is important to differentiate IgG4-RD from malignant tumors of each organ (e.g. cancer, lymphoma) and similar diseases (e.g. Sjögren’s syndrome, primary sclerosing cholangitis, Castleman’s disease, secondary retroperitoneal fibrosis, Wegener’s granulomatosis, sarcoidosis, Churg–Strauss syndrome) by additional histopathological examination.  Patients who cannot be diagnosed using the comprehensive criteria may be diagnosed using organspecific diagnostic criteria for IgG4-RD. |

Supplementary Table 2 2011 pathology consensus

| **Histological diagnostic scheme of IgG4-related disease** | | |
| --- | --- | --- |
| 1. **Histological features** | | |
| 1. Dense lymphoplasmacytic infiltrate | | |
| 1. Fibrosis, usually storiform in character | | |
| 1. Obliterative phlebitis | | |
| **2. Numbers of IgG4+ plasma cells/high power field (IgG4/HPF)** | | |
|  | Cases with 2 histological features | Cases with 1 histological feature |
| Meningus | >10 | >10 |
| Lacrimal gland | >100 | >100 |
| Salivary gland | >100 | >100 |
| Lymph node | >100 | >50 |
| Lung (surgical specimen) | >50 | >50 |
| Lung (biopsy) | >20 | >20 |
| Pleura | >50 | >50 |
| Pancreas (surgical specimen) | >50 | >50 |
| Pancreas (biopsy) | >10 | >10 |
| Bile duct (surgical specimen) | >50 | >50 |
| Bile duct (biopsy) | >10 | >10 |
| Liver (surgical specimen) | >50 | >50 |
| Liver (biopsy) | >10 | >10 |
| Kidney (surgical specimen) | >30 | >30 |
| Kidney (biopsy) | >10 | >10 |
| Aorta | >50 | >50 |
| Retroperitoneum | >30 | >30 |
| Skin | >200 | >200 |
| **3. Ratio of IgG4+/IgG+ cells >40% is a mandatory for histological diagnosis of IgG4-RD** | | |

Gray box=Histologically highly suggestive of IgG4-RD

White box=Probable histological features of IgG4-RD

Cases outside the two categories described above are insufficient.

Supplementary Table 3 Histological Characteristics in 4 biopsy organs of classified as IgG4-RD

|  | Salivary gland (n=41) | Lymph node (n=8) | Retroperitoneum (n=9) | Lacrimal gland (n=6) | *P* |
| --- | --- | --- | --- | --- | --- |
| Storiform fibrosis, n (%) | 23 (56.1) | 1 (12.5) | 7 (77.8) | 2 (33.3) | **0.033** |
| Obliterative phlebitis, n (%) | 14 (34.1) | 2 (25.0) | 1 (11.1) | 3 (50.0) | 0.446 |
| IgG4/HP, Median (IQR) | 72.5 (42.5, 127.5) | 125.0 (85.0, 245.0) | 50.0 (44.0, 85.0) | 115.0 (87.5, 147.5) | 0.177 |
| IgG4/HP>10, n (%) | 37 (90.2) | 8 (100.0) | 9 (100.0) | 6 (100.0) | 1.000 |
| IgG4/IgG (%), Median (IQR) | 70.0 (59.2, 84.5) | 67.5 (85.0, 100.0) | 55.0 (36.7, 68.3) | 77.8 (51.7, 93.8) | 0.402 |
| IgG4/IgG>40%, n (%) | 35 (85.4) | 7 (87.5) | 8 (88.9) | 6 (100.0) | 1.000 |

Supplementary Table 4 Comparison of Needle Biopsy and Surgical Biopsy in 72 diagnosed IgG4-RD samples

|  | Needle biopsy (n=24) | Sugical biopsy (n=48) | *P* |
| --- | --- | --- | --- |
| Age, Median (IQR) | 56.0 (50.3, 63.5) | 56.5 (48.8, 64.8) | 0.684 |
| Gender, Male, n (%) | 14 (58.3) | 30 (62.5) | 0.732 |
| Serum IgG4 (mg/dl), Median (IQR) | 808.0 (157.0, 1890.0) | 770.0 (323.8, 1660) | 0.618 |
| IgG4-RD RI | 4.0 (2.3, 6.0) | 4.0 (2.0, 7.0) | 0.711 |
| IgG4/HP, Median (IQR) | 50.0 (35.0, 80.0) | 100.0 (52.5, 140.0) | 0.003 |
| IgG4/HP, n (%) | | | |
| > 10 | 18 (75.0) | 48 (100.0) | 0.001 |
| ≤ 10 | 3 (12.5) | 0 (0.0) | 0.034 |
| Indetermined | 3 (12.5) | 0 (0.0) | 0.034 |
| IgG4/IgG (%), Mean ± SD | 70.1 ± 26.0 | 66.2 ± 22.1 | 0.438 |
| IgG4/IgG, n (%) | | | |
| > 40% | 19 (79.2) | 43 (89.6) | 0.399 |
| ≤ 40% | 4 (16.7) | 5 (10.4) | 0.705 |
| Indetermined | 1 (4.2) | 0 (0.0) | 0.333 |
| Storiform fibrosis, n (%) | 11 (45.8) | 24 (50) | 0.739 |
| Obliterative phlebitis, n (%) | 5 (20.8) | 18 (37.5) | 0.153 |
| 2011 Comprehensive Diagnostic Criteria, n (%) | | | |
| Possible | 7 (29.2) | 4 (8.3) | 0.049 |
| Probable | 1 (4.2) | 13 (27.1) | 0.045 |
| Definite | 16 (66.7) | 31 (64.6) | 0.861 |
| 2011 Histological Diagnostic Criteria, n (%) | | | |
| Insufficient | 19 (79.2) | 11 (22.9) | <0.001 |
| Probable | 4 (16.7) | 14 (29.2) | 0.248 |
| Highly suggestive | 1 (4.2) | 23 (47.9) | <0.001 |
| Biopsy organ, n (%) | | | |
| Meningus | 0 (0.0) | 1 (2.1) | 1.000 |
| Lacrimal gland | 0 (0.0) | 6 (12.5) | 0.169 |
| Salivary gland | 18 (75.0) | 23 (47.9) | 0.029 |
| Lymph node | 1 (4.2) | 7 (14.6) | 0.255 |
| Lung | 2 (8.3) | 0 (0.0) | 0.108 |
| Pleura | 0 (0.0) | 1 (2.1) | 1.000 |
| Pancreas | 1 (4.2) | 1 (2.1) | 1.000 |
| Bile duct | 1 (4.2) | 0 (0.0) | 0.333 |
| Retroperitoneum | 1 (4.2) | 8 (16.7) | 0.256 |
| Prostate | 0 (0.0) | 1 (2.1) | 1.000 |
